# Loss of Kaiso expression in breast cancer cells prevents intra-vascular invasion in the lung and secondary metastasis

**DOI:** 10.1371/journal.pone.0183883

**Published:** 2017-09-07

**Authors:** Jacek M. Kwiecien, Blessing I. Bassey-Archibong, Wojciech Dabrowski, Lyndsay G. Rayner, Alexandra R. Lucas, Juliet M. Daniel

**Affiliations:** 1 Department of Pathology and Molecular Medicine, HSC 1U22D, McMaster University, Hamilton, Ontario, Canada; 2 Department of Clinical Pathomorphology, Medical University of Lublin, Lublin, Poland; 3 Department of Biology, LSB 331, McMaster University, Hamilton, Ontario, Canada; 4 Department of Anaesthesia and Intensive Therapy, Medical University of Lublin, Lublin, Poland; 5 Biodesign Institute, Arizona State University, Tempe, Arizona, United States of America; University of South Alabama Mitchell Cancer Institute, UNITED STATES

## Abstract

The metastatic activity of breast carcinomas results from complex genetic changes in epithelial tumor cells and accounts for 90% of deaths in affected patients. Although the invasion of the local lymphatic vessels and veins by malignant breast tumor cells and their subsequent metastasis to the lung, has been recognized, the mechanisms behind the metastatic activity of breast tumor cells to other distal organs and the pathogenesis of metastatic cancer are not well understood. In this study, we utilized derivatives of the well-established and highly metastatic triple negative breast cancer (TNBC) cell line MDA-MB-231 (MDA-231) to study breast tumor metastasis in a mouse model. These MDA-231 derivatives had depleted expression of Kaiso, a POZ-ZF transcription factor that is highly expressed in malignant, triple negative breast cancers. We previously reported that Kaiso depletion attenuates the metastasis of xenografted MDA-231 cells. Herein, we describe the pathological features of the metastatic activity of parental (Kaiso^positive^) versus Kaiso^depleted^ MDA-231 cells. Both Kaiso^positive^ and Kaiso^depleted^ MDA-231 cells metastasized from the original tumor in the mammary fat pad to the lung. However, while Kaiso^positive^ cells formed large masses in the lung parenchyma, invaded large pulmonary blood vessels and formed secondary metastases and large tumors in the distal organs, Kaiso^depleted^ cells metastasized only to the lung where they formed small metastatic lesions. Importantly, intravascular invasion and secondary metastases in distal organs were not observed in mice xenografted with Kaiso^depleted^ cells. It thus appears that the lung may constitute a barrier for less invasive breast tumors such as the Kaiso^depleted^ TNBC cells; this barrier may limit tumor growth and prevents Kaiso^depleted^ TNBC cells from invading the pulmonary blood vessels and forming secondary metastases in distal organs.

## Introduction

An estimated 90% of medical fatalities in cancer patients are due to metastases [1]. Carcinomas of the mammary gland [2,3], prostate gland [4], liver [5–9], pancreas [10], endometrium [11], thyroid gland [12,13] and Merkel cell [14] have been shown to invade lymphoid vessels and blood vessels [2,6,8,9,11–14] resulting in metastases to distant organs, particularly the lung [7,9]. According to the systemic and pulmonary circulatory patterns, cancer cells that escape the primary tumor site and enter the bloodstream would first disseminate to the lung via the right ventricle before dispersing from the lung through the left heart to distal organs. Characterization of the mechanisms of malignant mammary carcinoma indicates that in the primary tumor, a small population of cells travel towards the blood vessels, and invade them in a complex process involving enhanced activity of genes regulating the dynamics of the actin cytoskeleton, e.g. Mena [2,15–17] and LIM kinase1 [18]. In a series of *in vivo* experiments in mouse and rat models utilising multiphoton microscopy, migrating tumor cells were seen to be assisted by perivascular macrophages in the process of vascular invasion and metastases to distant organs, which involved chemotaxis regulated by EGF and CSF-1 [1,15–17,19–23].

The progression of breast tumors towards an invasive behaviour and metastasis are postulated to involve several molecular factors associated with the complex epithelial-to-mesenchymal transformation (EMT) process that endows tumor cells with the ability to modulate their cell-cell adhesion and the extracellular matrix [24–26,47,48,50–52], apparently involved in the first line of defense against metastatic tumors. EMT is frequently accompanied by loss of the epithelial marker E-cadherin, concurrent with elevated expression of vimentin [41–45], an intermediate filament that participates in cell motility [46], as well as increased expression of matrix metalloproteases-2 and -9 (MMP-2, MMP-9) [27–32] that are often assessed in the determination of poor prognosis in breast cancer patients [33–36]. Tissue plasminogen activator (tPA) and urokinase plasminogen activator (uPA) are known to activate pro-enzyme forms of MMP-2 and MMP-9 to active forms [37,38] and tPA and uPA have been proposed as markers for breast cancer progression [39,40].

Despite remarkable advancements in our understanding of cellular and molecular mechanisms involved in tumor growth and intravascular invasion at primary tumor sites, relatively little is known about how malignant tumors travel to distant organs. Since all lymph and venous blood from the body flows via the right heart ventricle to the lung, it is expected that most if not all primary metastases of carcinomatous tumors are trapped in small pulmonary blood vessels. However, the relevant questions regarding the pathogenesis of metastatic cancer remain; how do secondary metastases travel to other distant organs, and what are the mechanisms involved in the generation of these secondary metastases?

To gain insight into the mechanisms involved in breast tumor metastases to distant organs, we studied the pathogenesis of secondary metastases of parental, Kaiso^positive^ and experimental Kaiso^depleted^ MDA-231 TNBC cells in immunocompromised mice [53]. Kaiso is a dual-specificity transcription factor that is highly expressed in, and linked with the aggressive features of breast, prostate, colon and pancreatic carcinomas [54–57]. We previously reported that Kaiso depletion strongly inhibited the metastasis of TNBC cells to distal organs [53]. Herein, we describe histological analyses of the metastases generated by parental Kaiso^positive^ and Kaiso^depleted^ MDA-231 cells.

We found that Kaiso^positive^ MDA-231 breast cancer cells implanted into the mammary fad pad of immunocompromised mice [53] formed large tumor masses in the lung parenchyma, invaded large blood vessels and metastasised to other distant organs where it also formed large masses. In contrast, Kaiso^depleted^ tumor cells formed small clusters only in the lung parenchyma and did not invade blood vessels and did not metastasize to distant organs. While the role of high Kaiso expression in the metastatic activity of human breast cancer cells was reported in our previous paper [53], we describe here the critical importance of high Kaiso expression in the propagation of breast tumors to distal organs beyond the lung, which we call “secondary metastases”. We propose that the lung serves as the second line of defense against carcinomas with metastatic potential; tumors with less malignant characteristics such as Kaiso^depleted^ MDA-231 cells are trapped, do not progress and perhaps are eliminated. In contrast, malignant tumors such as Kaiso^positive^ MDA-231 cells thrive in the lung to form large masses that then, invade the large pulmonary blood vessels, travel via the left heart ventricle and lodge in small blood vessels of a variety of peripheral organs where they form secondary metastases.

## Materials and methods

### Generation of stable Kaiso-depleted MDA-231 cells

The MDA-231 TNBC cell line was purchased from the **A**merican **T**ype **C**ulture **C**ollection (ATCC) (Manassas, VA, USA), and cultured in Dulbecco’s Modified Eagle’s Medium (DMEM; Lonza BioWhittaker^®^, Walkersville, MD, USA) supplemented with 10% Fetal Bovine Serum (FBS; Hyclone, Logan, Utah, USA), 0.1 mg/mL penicillin/streptomycin and 0.25 g/mL Fungizone (Invitrogen, Grand Island, NY, USA). Cells were passaged every 2 or 3 days and grown in a 5% CO_2_ humidified incubator at 37°C. Stable Kaiso-depletion was achieved by using multiple Kaiso-specific **s**hort **h**airpin **RNA**s (shRNA) that target the Kaiso mRNA specifically as previously described [49]. A scrambled shRNA that does not target the Kaiso mRNA was used as a control. Stable control (Kaiso^positive^) and Kaiso^depleted^ (sh-K) MDA-231 cells were maintained in DMEM-supplemented media treated with Puromycin (Invitrogen) at 0.8 μg/mL.

### Western blot analysis

Stable control Kaiso^positive^ and Kaiso^depleted^ (sh-K1 and sh-K2) MDA-231 cells were cultured until ~80–90% confluent, washed twice with cold PBS, and then harvested by cell scraping into cold microfuge tubes. Control, sh-K1 and sh-K2 MDA-231 cells were then pelleted by centrifugation, lysed, and protein isolated as previously described [59]. Rabbit anti-Kaiso polyclonal (1:5,000 dilution; a generous gift from Dr. A. Reynolds), and mouse anti-β-actin monoclonal (1:50,000 dilution, Sigma Aldrich, Oakville, ON, Canada) primary antibody incubations were performed overnight at 4°C. Secondary antibody incubations were performed with goat anti-rabbit- or donkey anti-mouse-horseradish peroxidase-conjugated secondary antibodies. (1;10,000 dilution, Jackson ImmunoResearch Laboratories, West Grove, PA, USA). Signals were then amplified with Clarity Western Enhanced Chemiluminescence substrate as previously described [53]. The sh-K2 MDA-231 cells, hereafter referred as sh-K or Kaiso^depleted^ MDA-231 cells, where chosen for all subsequent studies as these cells displayed the most efficient Kaiso knockdown.

### Animal studies

All animal studies were approved and performed at McMaster University, Ontario, Canada according to the guidelines by the Canadian Council for Animal Care. Extensive description of animal studies has been outlined previously [53]. Briefly, 4.5 x 10^6^ Kaiso^positive^ or Kaiso^depleted^ MDA-231 cells were injected subcutaneously into the mammary fat pad of 6–8 week old female **N**OD **S**CID **G**amma (NSG, Jackson Laboratories) mice (n = 5 each per condition), and allowed to form prominent subcutaneous masses up to 3,300 mm^3^ in volume. Non-invasive monitoring of mice was performed once a week, and increased to 2–3 times per week upon tumor appearance. Tumor growth was monitored externally with vernier calipers and tumor volume (in mm^3^) measured using the following formula; length/2 × width^2^, 2–3 times per week [53]. This tumor mass volume was achieved before the onset of serious clinical signs such as body weight loss, dehydration and lethargy that would require the application of the endpoint and euthanasia. The endpoint tumor volume of 3,300 mm^3^ was determined in pilot experiments prior to the study. At endpoint, the mice were euthanized by overdosing with intraperitoneal injection of sodium pentobarbital (100 mg/kg body weight), perfused when deeply anaesthetized, and fixed in 10% formalin and all routine tissues collected for histological examination.

### Histology and Immunohistochemistry

Harvested and formalin-fixed tissues were processed, embedded in paraffin wax, and 5 μm thick tissue sections were then mounted on glass slides and stained with either hematoxylin & eosin (H&E) or Masson’s trichrome. Immunohistochemical (IHC) analyses of tissue sections were performed as described previously [53]. Briefly, tissues were rehydrated in decreasing concentrations of alcohol, and deparaffinized in xylene before antigen retrieval by heating tissues in a sodium citrate solution (pH 6.0) in a microwave. Primary antibody incubations were performed overnight at 4°C with the following antibodies: anti-Kaiso 6F mouse monoclonal (1:500), anti-Kaiso 12H mouse monoclonal (1:800) [58], anti-Vimentin rabbit monoclonal antibody (1:500; **C**ell **S**ignaling **T**echnology (CST), Danvers, MA, USA #5741), anti-E-cadherin mouse monoclonal antibody (1:50; BD Biosciences, Mississauga, ON, Canada 610182), anti-MMP-2 rabbit polyclonal antibody (1:1000; CST #4022BC), and anti-MMP-9 rabbit polyclonal antibody (1:1000; CST # 3852BC). Secondary antibody incubations were performed for 2 hours at room temperature with either biotinylated goat anti-mouse or donkey anti-rabbit antibody at a dilution of 1:1000. Negative controls were obtained by excluding primary antibody. Histological analysis of H&E, Masson’s trichome and IHC-stained tissue sections were performed using a Nikon Eclipse 50 light microscope and representative phenotypes photographed.

## Results and discussion

### Protein expression

**Fig 1:**
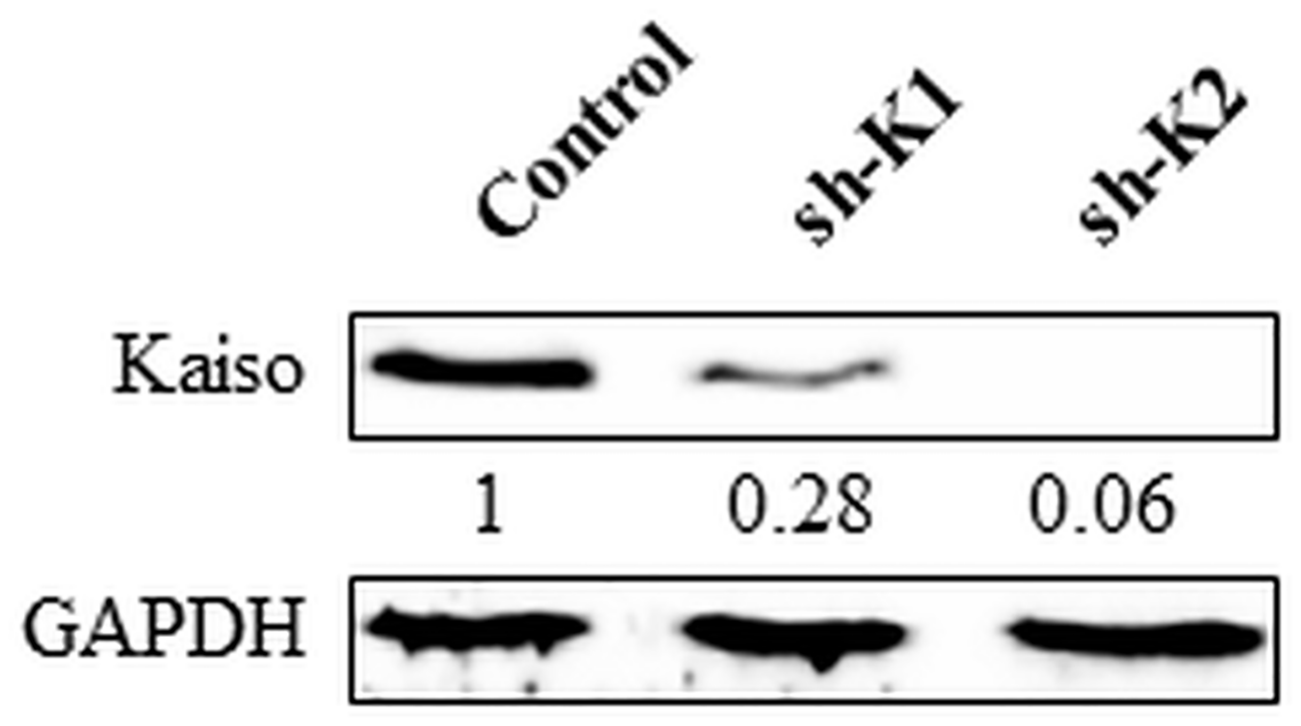
Kaiso^depleted^ MDA- 231 cells express negligible Kaiso compared to parental Kaiso^positive^ cells. Kaiso expression levels were determined using western blot. Both Kaiso^depleted^ clones (sh-K1 & sh-K2) expressed little Kaiso compared to the Kaiso^positive^ MDA-231 cells.

Expression of Kaiso in MDA-231 cells was abundant but remarkably reduced in sh-K1 (to 28%) and sh-K2 (to 6%) (see Fig 1) as the result of the stable transfection of the Kaiso-specific shRNA in these cells. Sh-K2 cells were utilized as Kaiso^depleted^ in the xenograft studies.

### Clinical observations and histological analyses

The subcutaneous masses in the mammary gland fat pad reached the endpoint volume of 3,300 mm^3^ within 8 weeks in Kaiso^positive^ tumors and 12 weeks in Kaiso^depleted^ tumors [60]. Further characterization of the Kaiso^positive^ and Kaiso^depleted^ MDA-231 phenotypes revealed that the difference in tumor growth was due to Kaiso-depletion effects on cell proliferation, anchorage-independence and apoptosis [60].

**Fig 2:**
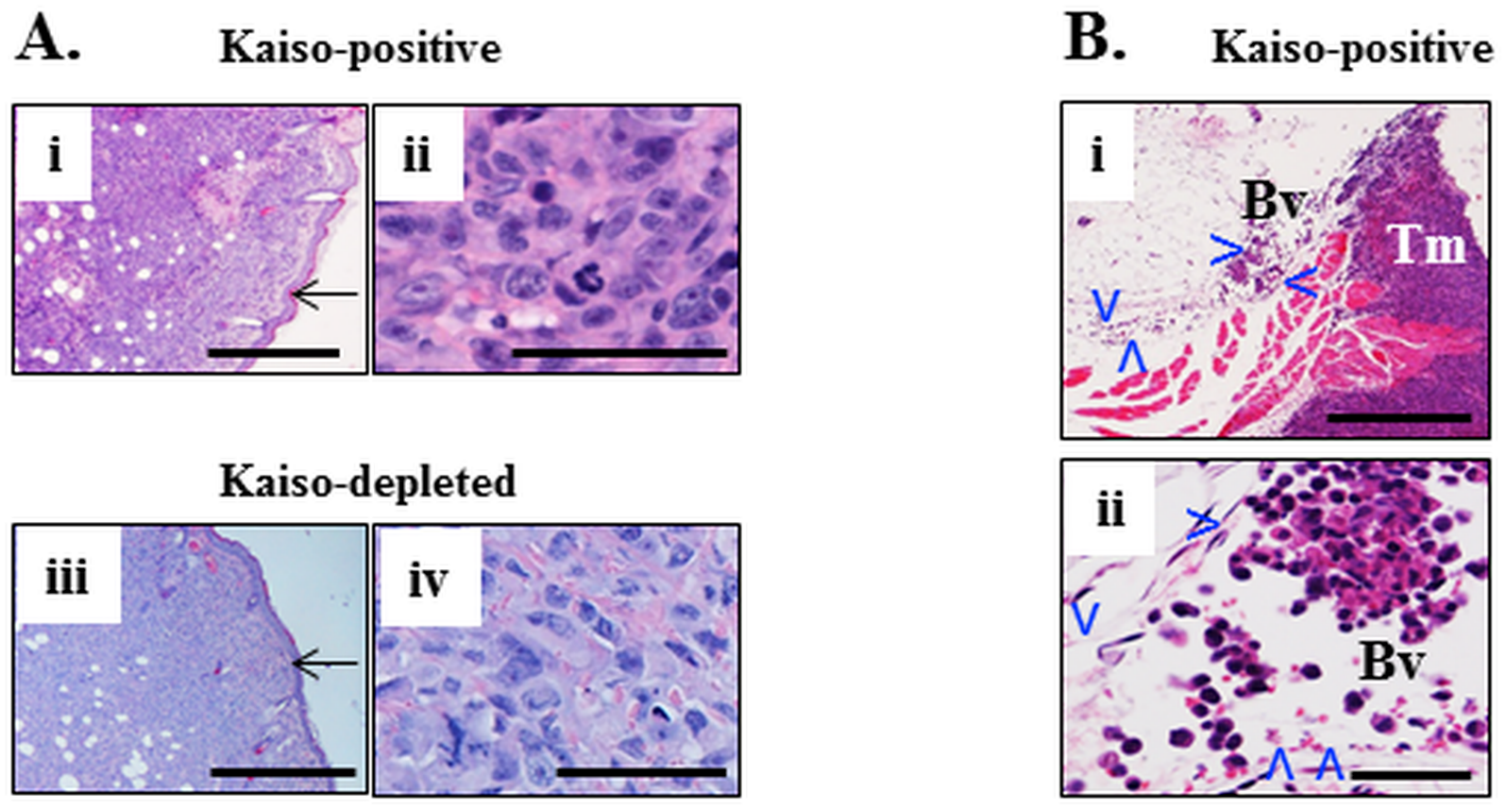
Primary subcutaneous tumors formed by Kaiso^positive^ and kaiso^depleted^ cells with invasion of the lumen of surrounding veins. Subcutaneous tumor mass of Kaiso^positive^ MDA-231 human mammary carcinoma cells (Ai, ii) and Kaiso^depleted^ tumor cells (Aiii, iv)) implanted into the fat pad of the mammary gland of female NRG mice. Tumor cells abut against the epidermis (arrow in Ai, iii) but do not invade it. Tumor cells are large, markedly pleomorphic, there is high mitotic index. (Bi) A vein (Bv, delineated by arrowheads) is adjacent to the subcutaneous tumor mass (Tm). It is distended by clumps and individual large pleomorphic cells (Bii) and also has scattered red blood cells. H&E. Size bars Ai, ii, Bi– 500 microns; Aii, iv, Bii– 50 microns.

The injection of the Kaiso^positive^ and Kaiso^depleted^ MDA-231 cells into the mammary fat pad of immunocompromised mice resulted in the formation of large subcutaneous masses (Fig 2Ai and 2Aiii) formed by large, pleomorphic cells with high mitotic index (Fig 2Aii and 2Aiv). Primary tumor masses formed by both types of mammary carcinoma cells were morphologically indistinguishable from each other (Fig 2A). Veins and lymphatic vessels in vicinity to some subcutaneous masses were distended with clusters or single tumor cells scattered throughout the lumen (Fig 2B).

**Fig 3:**
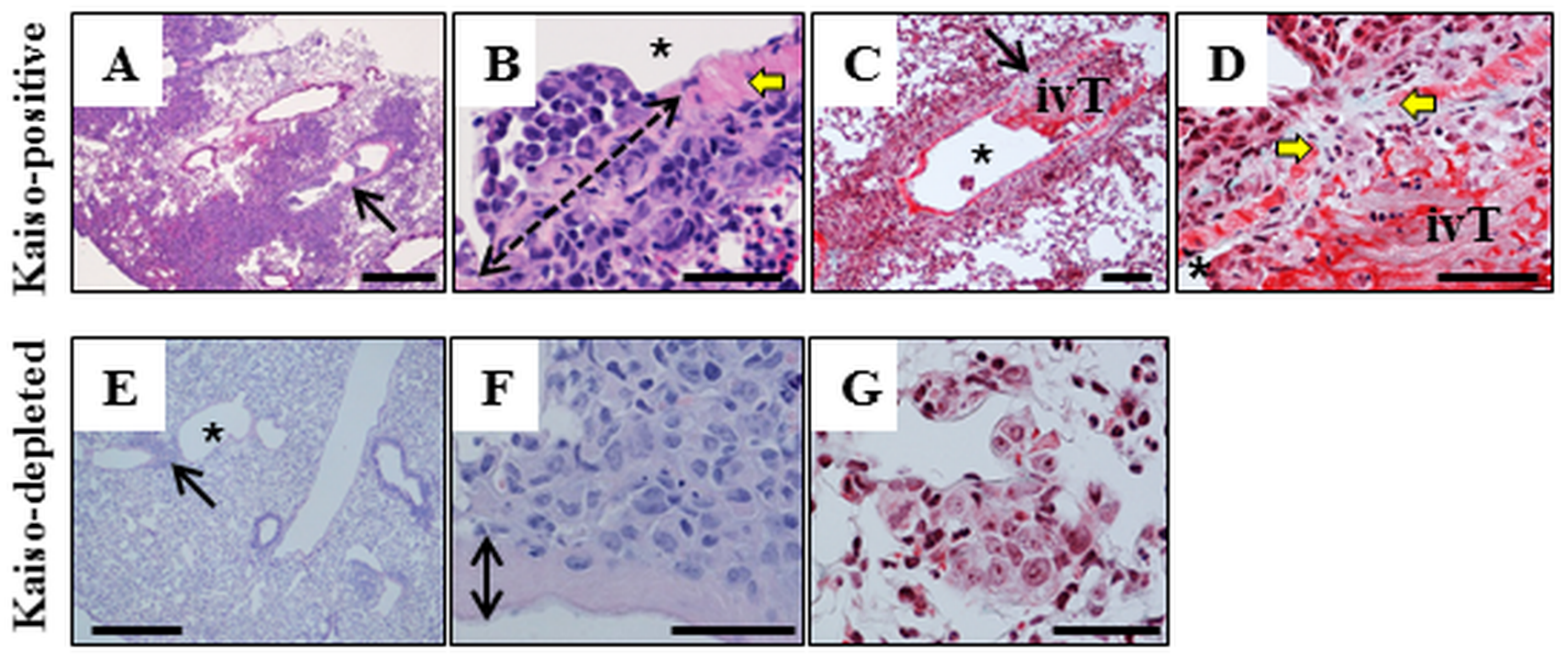
Metastasis of Kaiso^positive^ and Kaiso^depleted^ cells to the lung results in dramatically different tumor behavior. (A-D) Kaiso^positive^ tumors are numerous, large, obliterate the architecture of the pulmonary tissue and invade the lumen of large blood vessels (arrow in A). A segment of the vascular wall indicated by yellow arrows (B, D) is obliterated by tumor cells (double-headed interrupted arrow in B) that provide continuity between a perivascular mass and intravascular tumors (ivT) in the lumen (asterix in B, C). Apparent vascular invasion is associated with formation of intravascular thrombus (C, D). Intravascular surface of tumors or tumor thrombi is typically lined by endothelial cells (B). Tumor cells in intravascular masses or thrombi are large and pleomorphic (B, D). Kaiso^depleted^ cells form small interstitial aggregations (arrow in E) of large pleomorphic cells (F, G) that do not invade the wall or the lumen (F) of adjacent blood vessels. H&E–A, B, E, F; Masson’s trichrome (C, D, G). Size bars; A, E– 500 microns, B-D, F, G– 50 microns.

In the lung, Kaiso^positive^ cells formed large, often coalescing, non-circumscribed tumor masses with obliteration of the alveolar architecture (Fig 3A) formed by large, pleomorphic cells with a high mitotic index. Scattered neutrophils infiltrated the periphery of the tumor masses and the surrounding alveolar tissue. A proportion of large blood vessels encompassed by or adjacent to tumors (Fig 3A) had masses of tumor cells protruding into the lumen, with the segmental concurrent obliteration of the vascular wall by tumor cells forming a continuity of perivascular and intravascular tumors (Fig 3B–3D). The apparent vascular invasion of Kaiso^positive^ tumors was often associated by formation of thrombi infiltrated by tumor cells (Fig 3D). In contrast, Kaiso^depleted^ tumor cells formed small aggregations scattered in the alveolar parenchyma (Fig 3E and 3G), sometimes adjacent to large blood vessels but with no invasion of the vascular wall or the lumen (Fig 3F). Kaiso^depleted^ tumor aggregations were often infiltrated by scattered neutrophils (Fig 3G).

**Fig 4:**
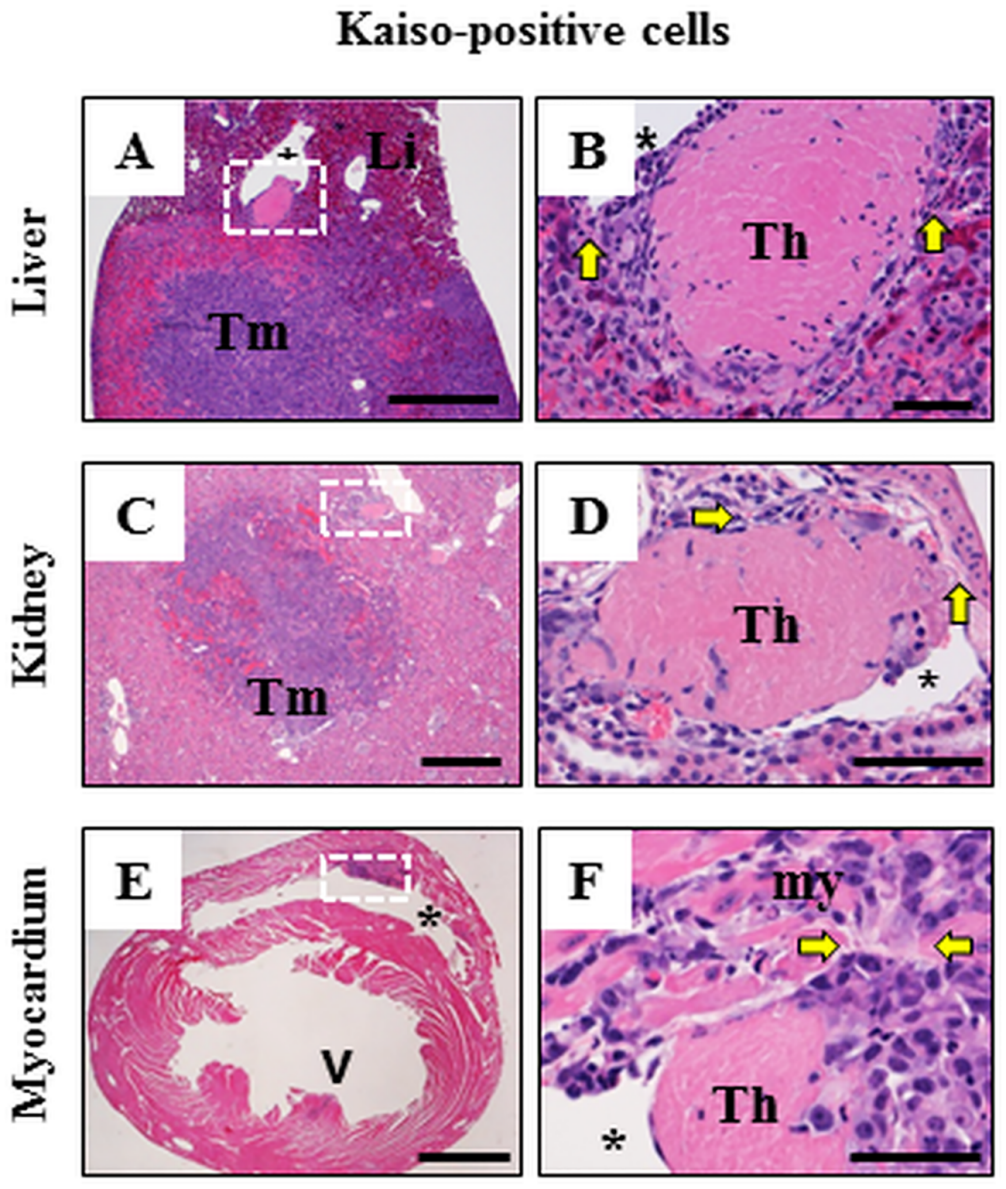
Intravascular invasion of secondary metastatic Kaiso^positive^ tumors. Low magnification images (A, C, E) and high magnification images (B, D, F) of tissue regions outlined by white dotted lines in A, C and E. Kaiso^positive^ cells metastatic to the liver (A, B) and kidney (C, D) formed large tumors and invaded adjacent blood vessels with formation of thrombi (Th in B, D) delineated from the surrounding tissue by yellow arrows. Tumor masses in the myocardium (white box and arrowhead in E) often resulted in invasion of the ventricle (asterix) with formation of a mass (white box in E) and thrombus (Th in F). Thrombus is delineated from myocardium (my) by yellow arrows in F. There is continuity between the masses of tumor cells in the myocardium and in the intraventricular thrombus (F). H&E. Size bars; A, C, E– 1,000 microns, B, D, F– 50 microns.

Although Kaiso^positive^ and Kaiso^depleted^ cancer cells both formed subcutaneous masses and invaded nearby veins and lymphatics resulting in pulmonary metastases, Kaiso-depletion markedly supressed the ability of cancer masses to grow expansively in the lung, and eliminated their ability to invade blood vessels thereby restricting their spread to other organs. Indeed, tumors were not observed in any other organ except in the lung of mice injected with Kaiso^depleted^ cells. In contrast, in mice injected with Kaiso^positive^ cells, large tumors were observed in the liver (Fig 4A and 4B), kidney (Fig 4C and 4D), myocardium (Fig 4E and 4F), and infrequently in the adrenal gland and leptomeninges of the brain (not shown). Thus, Kaiso appear to play a regulatory role to in the; (i) expansive growth of metastatic tumors in the lung and (ii) invasion of the pulmonary blood vessels to spread to other organs supports the notion of Kaiso as a crucial factor in highly aggressive subtypes of breast cancer [53,54,60]. Our findings suggest that Kaiso could be a target for therapeutic strategies in the treatment of aggressive breast cancers. Some large blood vessels within a tumor mass or adjacent to it, such as in the liver (Fig 4A), or in kidney (Fig 4C) had intraluminal invasion of tumor cells with formation of thrombus and obliteration of the adjacent segment of the wall of the blood vessel (Fig 4B and 4D). In the myocardium, protrusions of the tumor cells into the lumen of ventricles (Fig 4E) resulted in the formation of fibrinous thrombus (Fig 4F) infiltrated by tumor cells. Large, sometimes coalescing tumor masses obliterated the organ architecture and were composed of large pleomorphic cells similar morphologically and immunohistochemically to those described in the subcutaneous mass and in the lung of mice injected with Kaiso^positive^ cells.

**Fig 5:**
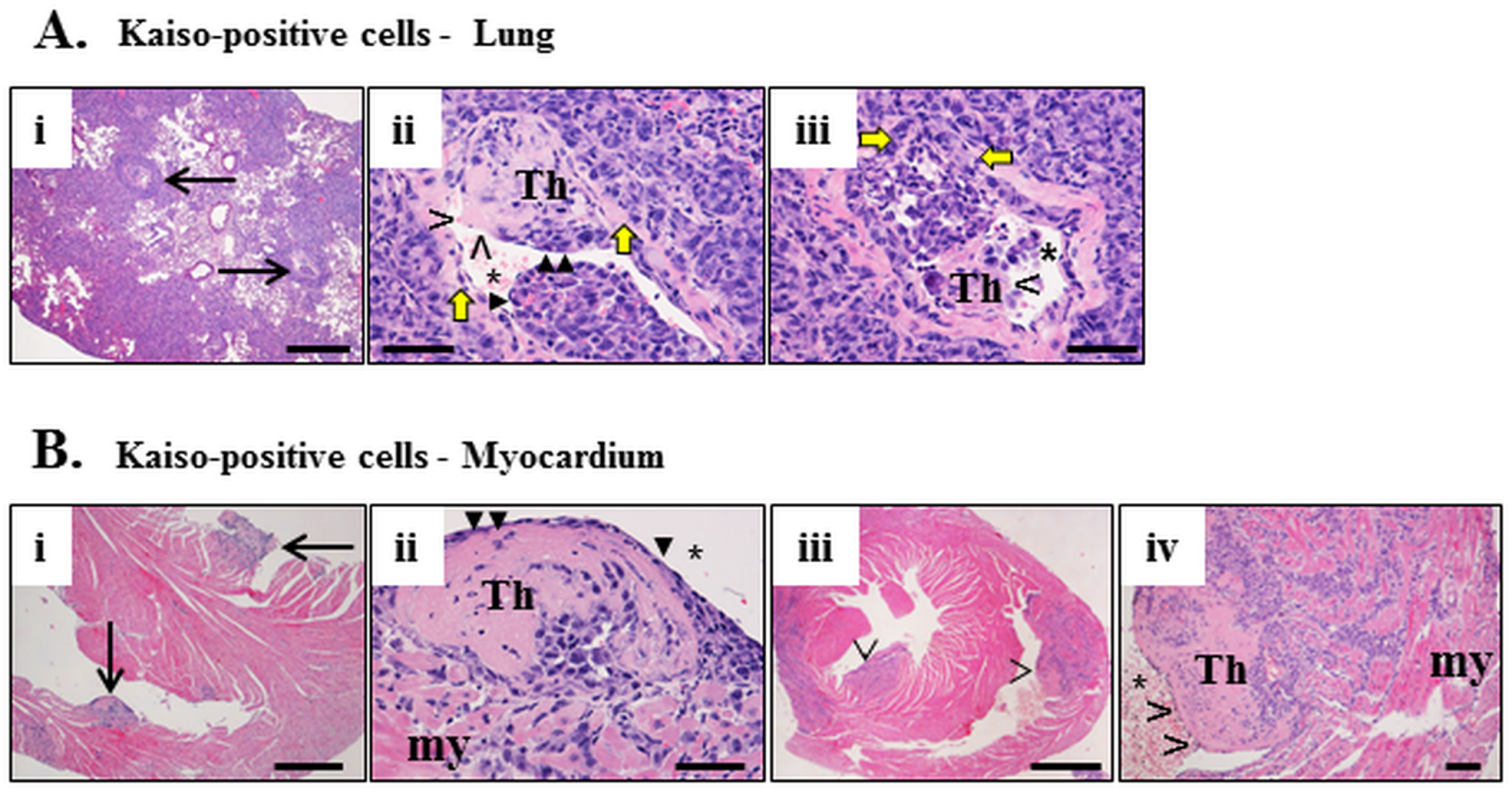
Thrombosis caused by Kaiso^positive^ tumors invading the blood vessels and heart ventricles. In the lung (A), a number of large blood vessels (two indicated by arrows) have intravascular thrombi delineated from the vascular wall by yellow arrows and protruding in the vascular lumen (Th in Aii, iii). The thrombi are infiltrated by neoplastic cells and are lined by endothelium (solid arrowheads in Aii) or not (open arrowhead in Aiii). In the myocardium (my, B) thrombi protruding into the ventricular lumen (Bi, iii) are also infiltrated by neoplastic cells (Th in Bii, iv) and either lined by endothelium (solid arrowheads in Bii) or not (open arrowheads in Biv). H&E. Size bars; Biii– 1,000 microns, Ai, Bi– 500 microns, Aii, iii, Bii, iv B– 50 microns.

Intravascular (Fig 5A) or cardiac intraventricular (Fig 5B) invasion by the Kaiso^positive^ tumor cells often resulted in formation of a thrombus whose surface in some cases was apparently covered by endothelium (Fig 5Aii and 5Bii), delineating the remaining lumen of the blood vessel or the ventricle. Other thrombi however, where not delineated by endothelium but often by a layer of neutrophils (Fig 5Aiii and 5Biv). In some blood vessels adjacent areas of thrombi were or were not endothelialized while in others apparently endothelium-free small clusters or individual cancer cells were present in the lumen (Fig 5Aiii).

The above data indicate that the subcutaneously implanted Kaiso^positive^ MDA-231 cells efficiently penetrated the vascular wall and invaded the lumen of large blood vessels in the lung and other organs (Figs 4 and 5). This is in line with other studies that have also demonstrated vascular taxis and intravascular invasion of breast tumors implanted subcutaneously into mice [1,15–17,19–23]. The active penetration of the vascular wall by Kaiso^positive^ cells lead to their accessing of the lumen with frequent formation of thrombus. This pathogenesis implies two potential mechanisms; (1) tumor cells breached the endothelium of the tumor–invaded blood vessel, which may have led to thrombosis; (2) the tumor cells then invaded the thrombus as the convenient substrate, which lead to the increase of the intravascular load of cancer cells destined to metastasize to other organs. We also observed endothelium lining of the intravascular tumor masses with or without thrombosis. We consider that neo-endothelialization of the intravascular tumor masses and tumor thrombi may serve as a defense mechanism preserving the patency of the blood flow. We thus postulate that this putative defense mechanism may actually be subverted by the invading tumor cells allowing for the increase of their intravascular load and presumably leading to a greater chance of success of secondary metastases.

### Immunohistochemical analyses

**Fig 6:**
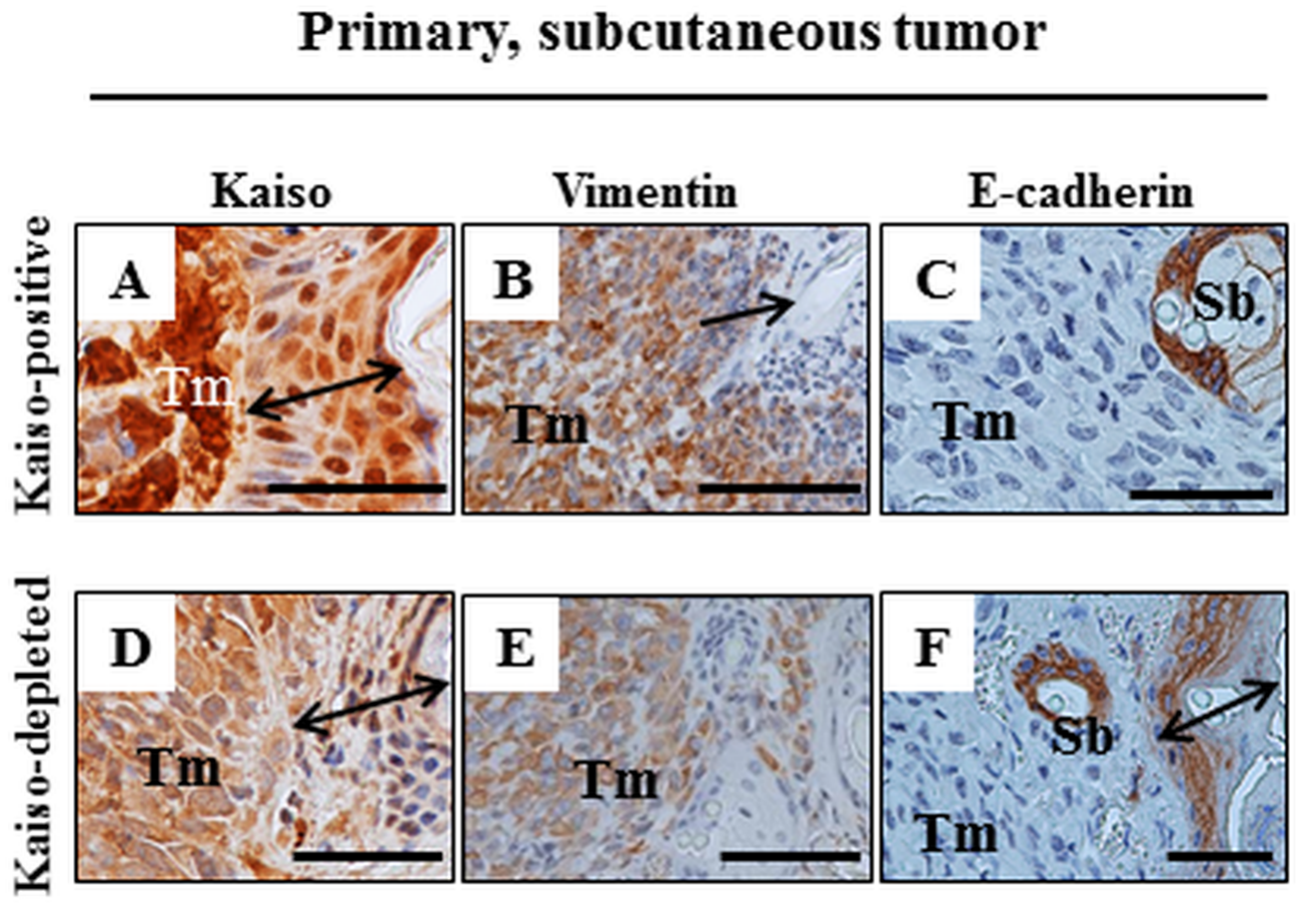
IHC of primary subcutaneous Kaiso^positive^ and Kaiso^depleted^ MDA-231 tumors. Tumor cells (Tm) of Kaiso^positive^ (A-C) and Kaiso^depleted^ (D-F) masses do not invade the epidermis (double-headed arrow in A, D, F, arrow in B). Kaiso^positive^ tumor cells are labeled strongly positive for Kaiso (A) and vimentin (B) while the Kaiso^depleted^ cells are labelled considerably less (D, E). The labeling with anti-E-cadherin antibody is negative for both types of tumor cells in contrast to the positive labelling of the mouse epithelium in sebaceous glands (Sb in C, F) and in epidermis (F). Size bars A-F– 50 microns.

Kaiso^positive^ primary tumor tissues, as expected, stained positive for Kaiso (Fig 6A), which localized to both the nucleus and cytoplasm. Further analysis of the Kaiso^positive^ tumor tissues for other molecular markers implicated in tumor metastasis revealed that similar to our previous observations *in vitro* [53], Kaiso^positive^ MDA-231 primary tumor tissues stained moderately for Vimentin (Fig 6B) and negative for E-cadherin (Fig 6C). We also examined the Kaiso^depleted^ primary tumor tissues for Kaiso, Vimentin and E-cadherin expression. While we expected little to no Kaiso staining in the Kaiso^depleted^ MDA-231 tumors as per our western blot results (Fig 1), we were surprised to observe weak Kaiso staining in the primary tumors which was predominantly cytoplasmic (Fig 6D). The weak Kaiso staining could be due to deselection of the Kaiso^depleted^ MDA-231 cells as the mice were not subjected to constant treatment with Puromycin that would ensure selection and maintenance of shRNA plasmid in the stable cells *in vitro*. Nonetheless, the Kaiso staining observed in Kaiso^depleted^ tumor tissues was still remarkably reduced compared to the staining observed in the Kaiso^positive^ tumor tissues (Fig 6A and 6D). Kaiso^depleted^ MDA-231 tumor tissues also stained weakly for Vimentin (Fig 6E) as expected from *in vitro* findings in our previous report [53]. In contrast, while we had observed increased E-cadherin expression in the Kaiso^depleted^ MDA-231 cells *in vitro* [53], the Kaiso^depleted^ MDA-231 tumor tissues stained negative for E-cadherin (Fig 6F). This lack of E-cadherin staining could be due to the weak levels of Kaiso expression observed in the Kaiso^depleted^ MDA-231 primary tumors (Fig 6D) or due to other *in vivo* factors in the tumor microenvironment that are independent of Kaiso’s expression or Kaiso’s effect on E-cadherin expression.

**Fig 7:**
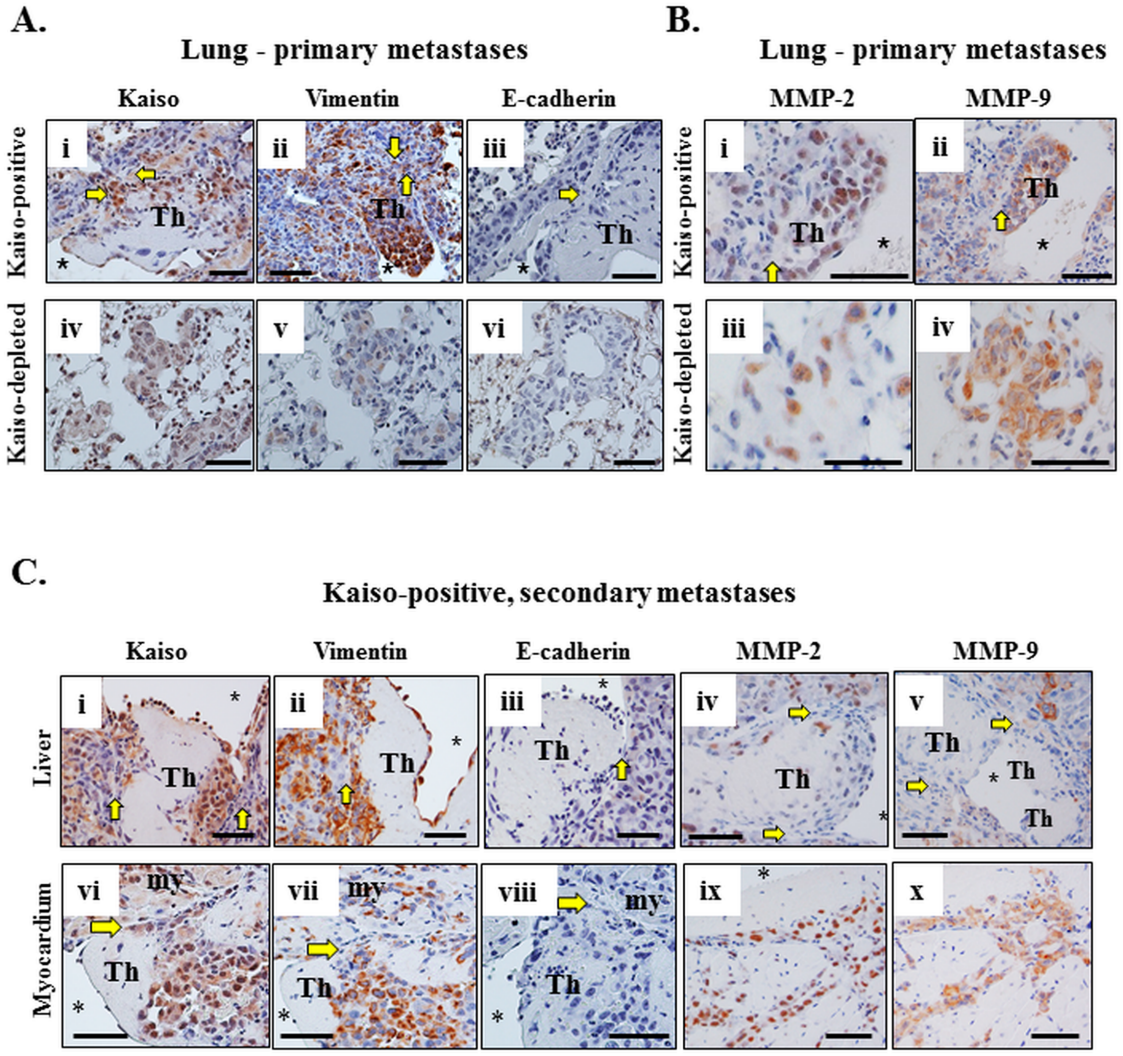
The molecular phenotype of the Kaiso^positive^ MDA-231 cells persist as they metastasize to other distal organs (liver and myocardium). (A) Neoplastic Kaiso^positive^ cells in lung metastases or thrombi are large, pleomorphic, and stain positive for Kaiso (i) and vimentin (ii), but negative for E-cadherin (iii). In contrast, Kaiso^depleted^ tumor cells are weakly stained for Kaiso (iv) and Vimentin (v) and negative for E-cadherin (vi). (B) Kaiso^positive^ tumor cells in lung metastases also stain strongly for MMP-2 (i), and MMP-9 (ii), while the Kaiso^depleted^ tumor cells stain weakly for MMP-2 (iii), but positive for MMP-9 (iv). The asterisk indicates the lumen of the blood vessel with the thrombus (Th) and the yellow arrows indicate the vascular wall and its obliteration by tumor cells in (A, B). (C) The thrombus (Th) formed in the large blood vessel of the liver (i-v) and in the ventricle of the heart (vi-viii) in mice injected with Kaiso^positive^ cells partially obliterated the vascular wall or endocardium indicated by the yellow arrows (i-viii). Neoplastic cells are stained positive for Kaiso (i, vi), vimentin (ii, vii), MMP-2 (iv, ix), and MMP-9 (v, x), and negative for E-cadherin (iii, viii). Scale bars; A-C– 50 microns.

Analysis of the tumor masses observed in the lung (Fig 7A) of mice injected with the Kaiso^positive^ and Kaiso^depleted^ MDA-231 cells also revealed that the Kaiso^positive^ tumor cells that metastasized to the lung exhibited strong Kaiso (Fig 7Ai) and Vimentin (Fig 7Aii) staining but no E-cadherin staining (Fig 7Aiii), while Kaiso^depleted^ tumor metastases in the lung displayed weak Kaiso (Fig 7Aiv) and Vimentin (Fig 7Av) staining, but no E-cadherin staining (Fig 7vi). These findings suggest a persistence of the molecular phenotype of both Kaiso^positive^ and Kaiso^depleted^ MDA-231 cells as they metastasized to the lung. We further analyzed the Kaiso^positive^ and Kaiso^negative^ MDA-231 lung metastases for the expression of MMP-2 and MMP-9. Although, MMP-2 and MMP-9 had not previously been examined in the Kaiso^positive^ and Kaiso^negative^ MDA-231 cells *in vitro*, they have been implicated in EMT and breast cancer progression [61–63]. Consistent with the EMT phenotype in the Kaiso^positive^ tumor cells, Kaiso^positive^ lung metastases displayed strong MMP-2 and MMP-9 expression (Fig 7Bi and 7Bii). In contrast, Kaiso^depleted^ lung metastases displayed reduced MMP-2 (Fig 7Biii) but not MMP-9 (Fig 7Biv) expression.

Examination of the Kaiso^positive^ tumor cells in secondary metastases (Fig 7C) also revealed strong Kaiso (Fig 7Ci) and Vimentin (Fig 7Cii) staining but no E-cadherin staining (Fig 7Ciii) in liver metastases. Similar to the lung metastases, the Kaiso^positive^ liver metastases also displayed strong MMP-2 (Fig 7Civ) and MMP-9 (Fig 7Cv) staining. Likewise, the Kaiso^positive^ tumor masses in the myocardium displayed strong Kaiso (Fig 7Cvi) and Vimentin (Fig 7Cvii) staining but no E-cadherin staining (Fig 7Cviii), as well as strong MMP-2 (Fig 7Cix) and MMP-9 (Fig 7Cx) staining. These findings also imply a persistence of the molecular phenotype of the Kaiso^positive^ MDA-231 cells as they metastasized to other distal organs.

In this study we analyzed the metastatic progression of Kaiso^positive^ and Kaiso^negative^ malignant mammary carcinomas using in vivo transplantation experiments in a mouse model. We found that Kaiso^positive^ and Kaiso^negative^ MDA-231 cancer cells both formed subcutaneous masses and invaded nearby veins and lymphatics apparently leading to pulmonary metastases. However, Kaiso-depletion was associated with remarkable suppression of the growth of cancer cells in the lung. In contrast, high Kaiso-expressing tumor cells thrived in the lung, invaded large pulmonary blood vessels and metastasized to other organs. These findings suggest that Kaiso plays a key role in metastatic activity of MDA-231 cancer cells. The penetration of the vascular wall and invasion of the lumen of large blood vessels abundant in the lung and other organs appears to be the fundamental factor of malignancy of the Kaiso^positive^ tumor cells. This is in line with other studies that have also demonstrated vascular taxis and intravascular invasion of breast tumors implanted subcutaneously into mice [1,15–17,19–23]. Immunohistochemical characterization of Kaiso^positive^ and Kaiso^depleted^ tumors in this study revealed that Kaiso^positive^ tumor cells exhibit more features associated with malignancy (increased Kaiso, Vimentin, MMP-2 and MMP-9 expression) than the Kaiso^depleted^ tumor cells, which only displayed increased MMP-9 expression. Moreover, Kaiso expression seem to correlate positively with Vimentin and MMP-2 but not MMP-9 expression. Indeed Kaiso-depletion resulted in decreased Vimentin and MMP-2 but not MMP-9 expression. The similar staining of MMP-9 and lack of E-cadherin staining in both Kaiso^positive^ and Kaiso^depleted^ tumor cells, which is a marker of EMT, may explain the ability of both cell types to metastasize to the lungs. However, considering that only the Kaiso^positive^ tumor cells were capable of surviving in the lungs, invading blood vessels and forming macrometastases in other distal organs, the higher Kaiso expression in concert with the increased Vimentin and MMP-2 expression could be considered as the critical determinants that allowed the Kaiso^positive^ tumors to thrive after metastasis to the lung, and other distant organs.

**Fig 8:**
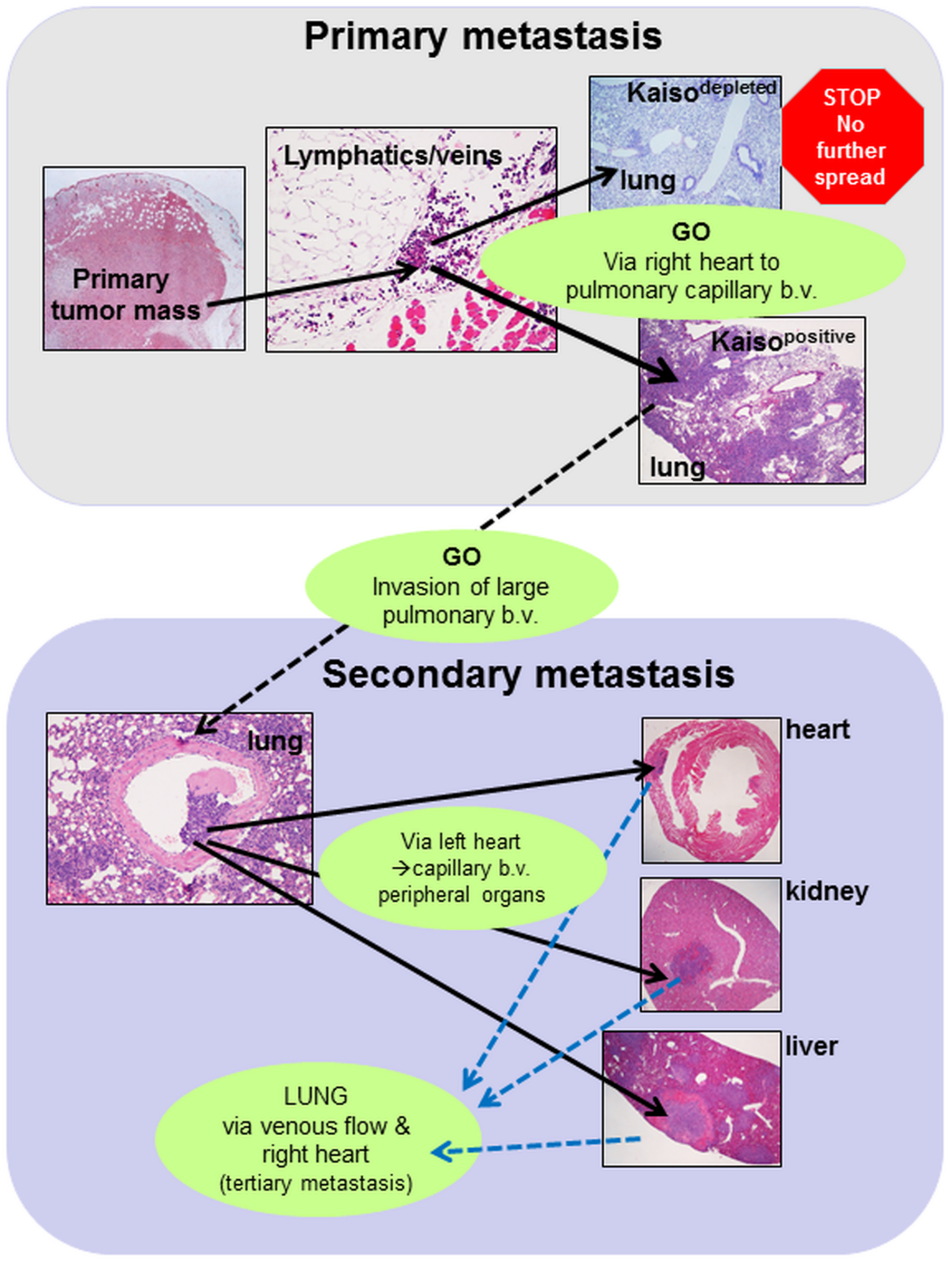
Conceptual pathogenesis of cancer metastasis. ***Primary metastasis***: Invasion of Kaiso^positive^ and Kaiso^depleted^ mammary carcinoma cells of the local veins and lymphatics allows the cells to migrate via the right heart ventricle to the lung where they are trapped in the capillary blood vessels (b.v.) and form pulmonary metastases. While in the lung, Kaiso^positive^ cells proliferate successfully and form large, coalescing masses that send the cells to actively cross the wall of adjacent blood vessels and invade their lumen. Kaiso^depleted^ tumor cells form small aggregations that do not invade blood vessels therefore the secondary metastases do not occur. ***Secondary metastasis***: The intravascular invasion by the Kaiso^positive^ tumor cells in the lung presumably leads to its migration in the blood via the left heart to a variety of organs notably heart, liver and kidney, where they form metastases and tumors with the invasion of local blood vessels or heart ventricles in a fashion similar to that observed in the lung. This may lead to tertiary metastases; via the venous flow to the right heart and ultimately to the lung.

We thus propose that the lung serves as the second line of defense against carcinomas with metastatic potential where tumors with less malignant characteristics, e.g. with reduced expression of Kaiso, are trapped, do not progress and perhaps are eliminated. Consequently, secondary metastases to the distant organs are prevented. We further postulate, that malignant tumors such as human breast tumors with high Kaiso expression can overcome this defensive mechanism, thrive in the lung and form large masses whose cells invade the blood vessels, travel via the left heart ventricle to lodge in small blood vessels of a variety of peripheral organs, and initiate multiple secondary metastatic tumors leading to accelerated demise (Fig 8). If this hypothesis is proven to be correct, potential cancer-suppressive tissue mechanisms in the lung should be considered in the pathogenesis of cancer metastasis. Also, models used to evaluate the effectiveness of anti-cancer therapies should specifically include the analysis of the primary metastases in the lung and secondary metastases from the lung to other organs. The possibility of the lung as the second line of defence, with potential anti-cancer mechanisms sufficient to stall Kaiso^depleted^ but not Kaiso^positive^ breast cancer cells should be addressed in further studies.

## Conclusions

In this report, we analyzed the metastatic progression of Kaiso^positive^ and Kaiso^negative^ malignant mammary carcinomas using *in vivo* transplantation experiments in a mouse model. Although this study utilizes the end point metastasis analysis of disseminated breast tumor cells, it highlights potential novel mechanisms involved in secondary metastases and provides detailed histological evidence of different behaviour of MDA-231 malignant breast cancer cells depending on the expression level of Kaiso. Both Kaiso^positive^ and Kaiso^depleted^ tumor types; (1) formed subcutaneous masses of cells with morphological features of malignancy; (2) invaded adjacent veins and lymphatic vessels; and (3) metastasized to the lung. However, while Kaiso^positive^ cells; (i) formed large pulmonary tumors; (ii) actively invaded pulmonary blood vessels apparently leading to (iii) secondary metastases and tumors in a variety of distal organs, Kaiso^depleted^ tumors formed only small aggregates in the lungs, did not invade pulmonary blood vessels and did not form secondary metastases. Thus, Kaiso may be a potent factor enabling breast cancer cells to overcome apparent inhibitory mechanisms in the lung and to send secondary metastases throughout distant organs.

## Supporting information

S1 Checklist ARRIVE.Guidelines. (PDF)
